# Assessing the impact of COVID-19 on the performance of organ transplant services using data envelopment analysis

**DOI:** 10.1007/s10729-023-09637-4

**Published:** 2023-04-26

**Authors:** Márcia N. F. Manoel, Sérgio P. Santos, Carla A. F. Amado

**Affiliations:** Faculty of Economics and Center for Advanced Studies in Management and Economics, University of Algarve, Faro, Portugal

**Keywords:** Organ donation-transplantation process, COVID-19, Performance assessment, Data envelopment analysis, Malmquist productivity index

## Abstract

Organ transplant is one of the best options for many medical conditions, and in many cases, it may be the only treatment option. Recent evidence suggests, however, that the COVID-19 pandemic might have detrimentally affected the provision of this type of healthcare services. The main purpose of this article is to use Data Envelopment Analysis and the Malmquist Index to assess the impact that the pandemic caused by the novel coronavirus SARS-CoV-2 had on the provision of solid organ transplant services. To this purpose, we use three complementary models, each focusing on specific aspects of the organ donation and transplantation process, and data from Brazil, which has one of the most extensive public organ transplant programs in the world. Using data from 17 States plus the Federal District, the results of our analysis show a significant drop in the performance of the services in terms of the organ donation and transplantation process from 2018 to 2020, but the results also indicate that not all aspects of the process and States were equally affected. Furthermore, by using different models, this research also allows us to gain a more comprehensive and informative assessment of the performance of the States in delivering this type of service and identify opportunities for reciprocal learning, expanding our knowledge on this important issue and offering opportunities for further research.

**Supplementary Information:**

The online version contains supplementary material available at 10.1007/s10729-023-09637-4.

## Introduction

Organ transplantation is a medical procedure in which an organ is removed from the body of a donor and placed in the body of a recipient to replace a damaged or failing organ. Undergoing an organ transplant can prolong a patient’s life and allow those with chronic illnesses to live a healthier life. Although many solid organs have been successfully transplanted, including the heart, kidneys, liver, lungs and pancreas, the process related to organ transplantation is complex and when performed poorly can have serious consequences not only in the access to care but also in the outcomes of the care provided. It becomes, therefore, increasingly important to foster improvement of the performance of transplant services aimed at making health services more accessible, efficient and effective. In order to do so, performance measurement of organ transplant services has become crucial to better manage resource allocation and cost reduction, whilst maximizing the quantity and quality of the services delivered and improving outcomes in organ donation and transplantation.

In recent decades, measurement of performance in the healthcare sector has expanded and methods such as Data Envelopment Analysis (DEA) and the Malmquist Productivity Index (MPI) have been widely used. By measuring performance, health organizations can assess whether they are progressing towards predetermined objectives, detect deviations from the plan and pinpoint areas of strengths and weaknesses [1]. Additionally, making comparisons among different health organizations—such as organ transplant services—offers important learning insights including the opportunity to reconsider and reformulate current service policies, health programs and initiatives in light of comparative evidence obtained through performance assessment [2].

While important progress has been made in organ transplantation in the last decades, with improvements in surgical methods, organ preservation and pharmaco-immunologic therapies [3], recent evidence suggests that the COVID-19 pandemic might have detrimentally impacted the provision of this type of healthcare services [4]. This is particularly concerning as it might have affected the number of those able to timely benefit from the clinical improvements achieved in organ transplantation.

This study is, therefore, justified on two grounds. Firstly, by the relevance of organ transplantation as a treatment for several medical conditions [5], the life-changing and often life-saving nature of this type of treatment, as well as the financial impact that organ transplant procedures have on healthcare systems worldwide. Secondly, by the need to obtain a comprehensive view of the performance of organ transplant services and assess the impact that the pandemic caused by the novel coronavirus SARS-CoV-2 had on the provision of these services.

Despite the clinical and financial relevance of the organ donation and transplantation (ODT) process, only a few studies have assessed its performance, and those who have studied this topic present some important limitations. In particular, they have focused primarily on a single type of transplantation, mostly kidney transplants; have not carried out an in-depth analysis of the ODT process and its glitches, or have not considered some important indicators in the analysis (e.g. number of patient survivors and transplantation waiting lists). Furthermore, to the best of our knowledge, no studies have yet been published exploring the use of nonparametric performance measurement techniques to assess the impact of the COVID-19 pandemic on the provision of these services.

By using Data Envelopment Analysis and data from 2018 to 2020, this research aims to evaluate the performance of solid organ transplant services pre and during the COVID-19 pandemic. In addition to providing an updated overview of the performance of the ODT process and exploring the impact of COVID-19 in the sector, the models proposed and the types of organ transplants included in the analysis are also different from the ones used in previous studies allowing for a more comprehensive and informative assessment of performance. In this respect, this study fills in an important gap in the literature regarding solid organ transplantation. The study also assesses the impact of the scale of operations and of the geographic variations on the access and quality of the ODT services delivered. Finally, it offers results and insights regarding the best performers. These results and insights can be useful to learn how to make a better use of financial and human resources, infrastructure and processes and, ultimately, assist health professionals, managers and policymakers’ better plan resource allocation.

Brazil was chosen as a case study because the Brazilian public health system has one of the most extensive public organ transplant programs in the world, funding more than 90% of transplants and offering full coverage of all the costs involved, from organ donation to post-transplant follow-up [5, 6]. Although the research focuses on the Brazilian case, the models we propose to assess the performance of the organ transplant services and the insights generated from the analysis, have also the potential to be applied in other countries and inform policy making at national and international levels.

In order to achieve the objectives above, the remainder of this article is organized as follows. First, we provide a brief background of the ODT process and of the Brazilian public health system, with particular emphasis on its organ donation-transplantation services. Then, we briefly discuss the relevance of performance measurement in the health sector and introduce the general aspects of the DEA technique and MPI. The previous studies that have used these techniques to assess ODT services are then reviewed. Next, we present and justify the models used in this study and discuss their results. Finally, we offer some suggestions for further research and some concluding remarks.

## Background and literature review

### The ODT process

Organ transplantation consists of a surgical procedure for removing organs from a donor—which can be living or deceased – to transplant these organs into one or more patients—recipients—who need this replacement treatment in order to restore the functions of diseased organs [7]. This treatment may represent the only possibility of therapy for many diseases and end-stage organ failure, having a great impact on the length and quality of life of patients.

Although organ transplantation has become a fundamental pathway in the management of severe organ failure worldwide, the ODT process is complex and involves many stakeholders. Not only is the process different between living and deceased donors, but it can also vary from country to country. Independently of the process adopted, there are some aspects that are similar. To begin with, as pointed out by Souza et al. [8: 208], “the donation of organs in death is only possible if brain death is diagnosed”. Neurological exams are therefore carried out on the potential donors to prove brain death before organ donation takes place. In addition, these patients usually need to be in Intensive Care Units (UCI) as specific medical devices are required to maintain artificial breathing and hemodynamic functions. This is necessary to prevent cardiac arrest and ensure blood circulation [9] in order to “maintain adequate conditions for the organs to be subject to donation” (Garcia [10: 23]). Then, the next step in the process is to obtain consent for organ donation. This is the part of the process where the greatest differences occur from one country to another, probably, due to the legislation that governs each country. Once consent for organ donation has been given, logistical arrangements have to be made for organ transplantation to take place. In particular, health care professionals need to evaluate the viability of the organs, materials are sent for immunology exams, waiting lists of potential organ recipients need to be consulted, donors’ surgeries need to be scheduled, removal teams and transplantation teams need to be arranged and, finally, the transport of the donated organs needs to be organized [10, 11]. After that, the final steps can then take place, which include the removal of the organs, the allocation of suitable organs according to the waiting list, the transplantation of the organs to the recipients and the follow-up of the results.

Adding to the existing obstacles to a smooth organ transplantation process, from 2020 onwards, other important barriers emerged, due to the novel coronavirus SARS-CoV-2, which implied targeting surgical centers for the care and hospitalization of COVID-19 patients, problems in transporting organs between hospitals, and reduced availability of medical supplies needed to perform transplants [12]. As stated by Doná et al. [13: 3], “the emergence of COVID-19 has had a profound impact on transplantation worldwide, with respect to not only issues around donors or recipients, but also healthcare resource utilization”. Consequently, health services worldwide had to redesign the way services were offered to patients, new protocols had to be incorporated to ensure the viability of the donated organs and measures had to be implemented to prevent infection by COVID-19 among recipients and health professionals, thus reducing the number of transplants performed and increasing the time on the waiting list. This research, in addition to assessing the performance of one of the most extensive public organ transplant programs in the world, also aims to shed more light on the impact of the COVID-19 pandemic on the provision of these services.

### The Brazilian public health system and its organ donation-transplantation services

Brazil is “considered to be the only country with a population of more than 200 million people to have a universal health care system” (Donida et al., [14: 2]), often designated by Unified Health System or “*Sistema Único de Saúde*” (SUS). The SUS is one of the most complex public health systems in the world due to its reach and multiplicity of health services [15]. Consequently, some of its programs and initiatives have been largely recognized as an international reference and have been studied, such as the programs on tobacco control, AIDS and organ transplantation [16, 17].

In fact, Brazil has the most extensive public organ, tissue and cell transplant program in the world, which is guaranteed to the entire population by the SUS and ranks second in the absolute number of transplants performed, behind the United States [5, 7]. In 2020 alone, 5833 solid organ transplants were performed, of which 3813 were kidney transplants [18]. Despite the large volume of surgeries performed in Brazil, the number of people on the waiting list to receive an organ is still large [7]. The total number of patients on the active patient waiting list in December 2020 for solid organs amounted to 28658 people, with 12757 new entries and only 31% of potential donors becoming effective donors [19].

In Brazil, organ donation from the deceased can only take place after the donor's brain death and with the family's authorization [7]. In 2020, 42% of family members refused to donate organs [19]. A low level of organ transplants can, therefore, indicate underutilization of potential donated organs, which can occur due to the inefficiency of the donation process or due to the low rate of organ donation approval by the families of the potential donors [5]. The low availability of organs can result in long waits for a transplant, in patients dying while they wait for organs or in patients being removed from the transplant list due to the deterioration of their clinical status [9, 20]. One factor that contributes to the “growing waiting list is inefficient management of available organ supply” (Marinho and Araujo [21: 570]). As pointed out by Marinho and Araujo [21: 574], “although the states cannot directly control the number of transplants and of brain death notifications, they are responsible for managing all available resources to ensure that donor conversion occurs”.

Currently, there are 586 establishments authorized for the removal of organs and tissues in Brazil and 380 establishments authorized to perform solid organ transplants [18]. Regarding the distribution of these establishments among States, the vast majority of transplant centers are located in the Southeast (190) and Northeast regions (78), followed by the South (73), Midwest (30) and the North regions (9) [18]. Despite the contribution of the SUS in expanding access to health services and improving the health of the population, it is recognized that there are still real inequalities in access to health services across the country [22]. By measuring the performance of the Brazilian states in delivering organ transplant services and in handling the COVID-19 pandemic, we aim to assess the extent of these inequalities and, through the identification of best practice states, offer insights that can potentially inform the design of better health policies.

### Performance measurement in the healthcare sector and the DEA technique

In this research, the term performance is employed with the meaning attributed to it by Cylus and Smith [23], who acknowledge that performance in the health sector is a general term to describe the success of healthcare delivery, which can take into account efficiency as well as other facets of a health system assessment. The performance of health systems can, therefore, be assessed with reference to a broad range of indicators relating to inputs (resources), outputs (results) and outcomes (impacts). In addition, these indicators can relate to many different aspects of the health system under observation (e.g. access, efficiency, effectiveness, equity, responsiveness and quality). All these aspects can make it difficult for stakeholders to objectively analyze the multitude of indicators available, which has led to the use of composite indicators in order to obtain an overall view of the performance of organizations [24]. Composite indicators combine separate performance indicators into a single index or measure, and are used to compare the performance of different professionals, organizations or systems, providing a more complete view of performance [24].

One technique broadly used in the health field to incorporate into a single metric a wide variety of variables without requiring the specification of a functional form relating the performance to its attributes, is the DEA technique. According to Cordero et al. [25] and Worthington [26], these are some of the reasons why most studies that measure the efficiency and the productivity of health institutions have chosen this method rather than parametric methods.

DEA is a nonparametric technique for analyzing the efficiency of services and organizations [27]. The DEA method was created by Charnes, Cooper and Rhodes in 1978, and the pioneering model is known as the CCR model. This programming technique can use multiple inputs and outputs without requiring preassigned weights and comprehends both technical and scale inefficiencies [28]. As summarized by Hamzah and See [29: 464], the “efficiency of a decision making unit is measured as the ratio of the sum of its weighted outputs to the sum of its weighted inputs”. In 1984, Banker, Charnes and Cooper extended the CCR model, originating the BCC model [28].

The basic idea behind the DEA technique is to determine the relative efficiency levels of different units—named decision-making units (DMUs) by constructing an ‘efficient frontier’ which envelops all inefficient DMUs and indicates the best practices [30, 31]. An efficiency score ranging from 0 to 100% is assigned to each DMU by measuring its distance from the efficient frontier. “Units which lie on the frontier are said to be 100% efficient, while others which are away from the frontier are inefficient, with efficiency scores below 100%” (Safdar et al., [31: 3]). The most efficient DMUs become benchmarks for inefficient units [32]. After solving the DEA model, for each inefficient DMU it is possible to identify its benchmarking group; that is, a group of units that are following the same objectives and priorities but performing better. Another interesting feature of this method is that it allows the calculation of target values, in terms of the inputs and outputs, for the inefficient units to reach the efficiency level [31].

Among the advantages of using DEA are the possibility of using multiple inputs and outputs without the analysis becoming too complex [32] and the fact that it does not require the stringent model testing typical of the statistical techniques [23].

Another important characteristic of the DEA method is that it can be used to measure changes in productivity over different periods of time using the MPI [25, 33], which is an index obtained by multiplying two indices: the ‘catch-up index’ and the ‘frontier-shift index’.

However, the use of the DEA technique in the health care context also shows some significant limitations as it assumes that it is possible to fully characterize the production of health care by identifying a set of inputs, outputs and outcomes of production. Nevertheless, as pointed out by Amado and Santos [34], some of these outputs and outcomes are not easily measurable. The analysis can also be vulnerable to data errors. If the data of an efficient DMU is incorrect, this can negatively influence the result of many of the inefficient DMUs [23]. As highlighted by Amado and Santos [34: 46], “awareness of these limitations and of their potential impact on the results is necessary if useful information is to be obtained”.

Please refer to the Appendix for a detailed explanation of the DEA method and of the MPI, as well as for the mathematical formulations associated.

### The related studies on the use of DEA

Due to the complexity of services provided by hospitals and the high financial volume invested by governments, the interest in evaluating the performance of healthcare services has increased considerably in recent years, with a review of the literature showing that DEA has been one of the most frequently used techniques for this purpose. For a recent review of applications of DEA to the healthcare sector, the reader is referred to Kohl et al. [30].

In regard to the use of DEA to assess the performance of organ transplant providers, the literature is very scarce though. In fact, to the best of our knowledge, only a very few studies have been documented. The first study in this context is that by Ozcan et al. [20], who used this methodology to benchmark organ procurement organizations based on the level of technical efficiency.

In Brazil, one of the first studies to measure the efficiency of organ transplantation was undertaken by Marinho and Cardoso [35], who evaluated the technical efficiency and scale efficiency of the Brazilian National Transplant System (NTS) from 1995 to 2003. The results demonstrated a reduction in the efficiency of the NTS during the period, with recovery between 2001 and 2003 and a variation in efficiency related to different types of transplants was also observed. Costa et al. [36], in turn, measured the efficiency in the public kidney transplant system across Brazilian states and their productivity trends from 2006 to 2011. The results of this study showed that the states of the South and Southeast regions carried out organ harvesting and transplant activities more efficiently. The results also indicated a large variability between the states and the Federal District, pointing to disparities in the management of resources applied in this sector. This study also identified a change in the efficiency frontier from 2006 to 2011 but did not reveal progress in productivity. Similar results were found by Siqueira and Araujo [9], in a study using data from 2013 to 2015 that examined the technical and scale efficiency of Brazilian public services in kidney transplantation. This study also showed that states from the South and Southeast regions in Brazil performed better when compared to the poorest states from the North and the Northeast. The research also pointed to decreased efficiency during the aforementioned period and a lack of progress in efficiency in recent years. A different approach was adopted by Arteaga et al. [37], who used information from 485 kidney transplant patients from living donors as DMUs to determine the potential success of the transplantation process in a Spanish hospital. The most recent study in this area is, however, the one developed by Marinho and Araujo [21], who applied DEA and the bootstrap method to quantify the efficiency of the Brazilian states and of the Federal District in providing transplant services by converting potential organ donors into real donors in 2018. The study shows considerable variability in terms of the level of efficiency among states, which is in line with the results of the other studies mentioned above. It also indicates that it would be possible to increase by 45% the number of transplanted organs without increasing the pool of potential donors. This shows that the inefficiency of converting potential donors into real donors contributes to the insufficient offer of organs for transplantation. Table A in the Supplementary Material summarizes the information about the studies that have used DEA to evaluate the organ donation and transplantation process.

The studies above clearly demonstrate that the use of DEA to assess performance in the health sector can provide fundamental information to allow a better use and prioritization of limited health resources. However, the literature review also shows that, despite the widespread use of DEA in the health sector, there is still a gap related to the assessment of organ donation and transplantation services. In particular, there is a lack of studies including information on different types of solid organ transplants and considering multiple outputs. The present study addresses these limitations by using information regarding the transplant of different solid organs (i.e. kidney, liver, heart, lung, pancreas and simultaneous pancreas and kidney transplantation) and by using several output indicators. Furthermore, by disaggregating the ODT process into three fundamental parts (Access to Services; Organ Donation and Harvest; and Organ Transplantation) and looking at how the COVID-19 pandemic has impacted this process, the study allows a more comprehensive and informative assessment of the performance of the transplant services and provides new insights into this field.

## Empirical analysis

### The DEA models

The conceptual framework proposed in this study aims to encompass the dimensions considered most relevant in measuring the performance of the transplant service system in Brazilian states and elsewhere. These dimensions are related to the population's needs, service capacity, access to services, resources used, services delivered, quality of services and outcomes achieved.

The population's needs refer to the estimated requirements for transplants in the period under analysis and can be inferred by looking at the number of registrations on the transplant waiting list. The resources used by the organ donation and transplantation services include financial, physical and human resources; and the services delivered can be measured using indicators such as the value of hospital services provided, number of transplants performed, number of brain death diagnoses and/or number of complications treated due to the medical procedure. An indicator of access to ODT services can be the number of establishments authorized to perform transplants and one related to the capacity of the service can be the number of transplant teams. Regarding the quality of the services provided, one classic indicator is the hospital mortality rate [38]. Finally, the health outcomes achieved should measure the change in the health status of the patient attributed to the medical intervention. In other words, they measure the impact of the transplant on the patient’s life which can be physical or psychological and related to the quantity and quality of life. As emphasized by Amado and Santos [34: 47], “the outcomes of care may be of a subjective nature, posing increased challenges for measurement” due to their characteristics. Based on these dimensions and on the performance indicators that they encompass, several measures of performance such as equity of access, efficiency and effectiveness can be developed.

**Fig. 1:**
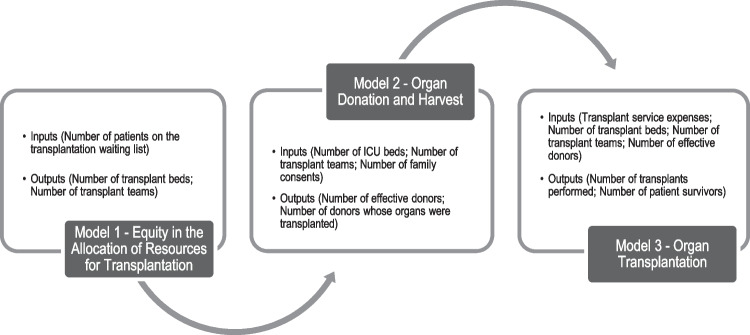
Conceptual Framework

The accuracy of the estimated performance measures depends on the use of appropriate and well-specified models, relevant variables and accurate data [39]. The analysis we propose uses three complementary models to evaluate the ODT process in Brazilian states. The first model focuses on the evaluation of the equity in the distribution of resources for transplantation. The second model is related to the organ donation and harvest process and the third model concerns the organ transplant service itself. Figure 1 shows the conceptual framework proposed, which lists the inputs and outputs used in the study.^1^

In this particular study, the selection of variables was based on our understanding of the ODT process and on previous studies in the area, and it took into account data availability. In particular, the outputs were chosen in consistency with the main objectives of the study. Then, we identified the inputs necessary to ensure the delivery of the chosen outputs.

Model 1 evaluates equity in the access to resources. In particular, this model tries to verify if the resources available are consistent with the needs of the population. In this respect, in model 1, the transplantation waiting list is used as an input related to population needs and the number of beds and transplant teams represent the essential resources required to ensure patient equity in obtaining the required transplants.

Model 2 seeks to assess the organ donation and harvest process. So, for this model, the input number of intensive care unit (ICU) beds represents the physical infrastructure necessary for organ transplantation to take place. According to Siqueira and Araujo [9], ICU beds are necessary for the identification and maintenance of deceased donors with brain death and are essential resources to reduce avoidable loss of potential donors and increase the number of brain death notifications. The other two inputs, number of transplant teams and number of family consents for organ donations represent, respectively, the human resources necessary for the service provision and, indirectly, the resources needed to perform transplants. Concerning the outputs, the number of effective donors represents the conversion of potential donors into real donors which is a necessary resource for a transplant to be performed [9]. The variable number of donors whose organs were transplanted represents the effective use of the organs that were harvested, in other words, it indicates that the organ was given to a compatible organ receiver on its ischemia time. Marinho and Araujo [21] have recently used this variable.

Model 3 assesses the last stage of the ODT process. In model 3, the input hospital and professional services expenses corresponds to the financial resources necessary to promote transplant surgeries. This variable has been previously used in the studies by Marinho and Cardoso [35] and Costa et al. [36]. The number of transplant beds and the number of transplant teams represent, in turn, the physical and human resources, respectively, while the number of effective donors represents the real capacity of the organ donation services. The number of transplants performed represents the ultimate output of the entire ODT process, which is directly linked to the other variables, and the number of patient survivors is the main goal of the organ transplantation service, as the objective is not just to perform the highest number of transplants possible to those who need them, but to ensure that the transplant recipients survive the procedure, as it will most likely lengthen and improve the quality of their lives. While the former variable has been frequently used (e.g. Siqueira and Araujo [9], Marinho and Araujo [21], Marinho and Cardoso [35], Costa et al. [36]), we found no evidence of the use of the latter.

The framework presented in Fig. 1 incorporates three production stages and may be seen as a network system. This is the case because one of the outputs from Model 1 (Number of transplant teams) is an input in Model 2. Similarly, one of the outputs from Model 2 (Number of effective donors) is an input in Model 3. For this reason, these two variables may be considered as intermediate products (as defined by Färe and Grosskopf [40]). Studying production processes as network systems can be advantageous when compared to the traditional black box approach (where a single model with the initial inputs and final outputs is considered), because it allows the identification of the production stages that require the most improvement.

There are several approaches that can be used to measure performance in network systems. One approach is to use the family of network DEA models, as initially proposed by Färe and Grosskopf [40], to endogenize the inner workings of the system. Another approach is to consider each stage as independent and measure the performance of each stage using standard DEA models [41]. By treating the stages as independent, this latter approach allows each stage to optimize the weights of the inputs and outputs independently, without imposing equality of the weight of the intermediate measures in the different stages where they appear. In his review, Kao [41] identifies several studies that have used the independent approach, such as, for example, the studies by Zhu [42] and Yang [43].

In this study, we use the independent approach because our objective is to measure the performance in each stage, in order to shed light on the causes and mechanisms behind poor performance in the organ transplant system. Despite recognizing the high relevance of network DEA, we do not consider it necessary to endogenize the inner workings of the system. In fact, in order to increase flexibility in the analysis, we consider preferable not to impose equality between the weights of intermediate products in the different stages of the organ donation process, allowing for the weights to comply with the defined production trade-offs in the specific stages.

**Fig. 2:**
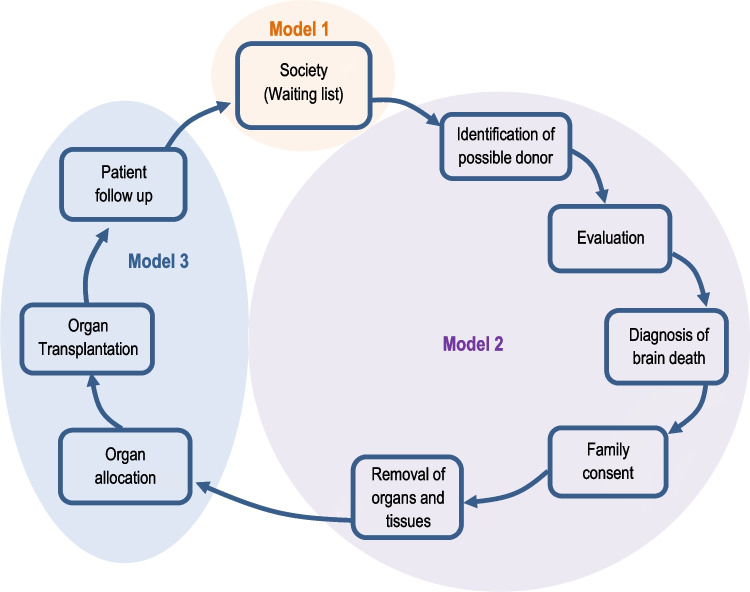
Steps of the organ donation-transplantation process and links between the three models. Source: Adapted from Garcia (Ed), 2017 [10]

The three models capture different steps of the process of organ donation-transplantation, as depicted in Fig. 2. By using three separate models, it is possible to capture three different criteria to evaluate the performance of the transplant services. Whilst model 1 evaluates if the available resources in a state are enough to satisfy population needs (measured by the people on the waiting list), model 2 evaluates if the resources available are being used to maximize the number of effective donors and the number of donors whose organs are transplanted. Finally, model 3 evaluates the effectiveness of the transplantation services by maximizing the number of transplants performed and the number of survivors. In order to achieve success in transplant services, it is important to have good performance in all three models. Insufficient access to resources (model 1) compromises organ-harvesting capacity (model 2) and poor harvesting compromises transplant services (model 3). As discussed by Gómez et al. [44: 3], once “a potential donor is identified, medical and surgical teams have to work quickly to extract usable organs and keep the *cold ischemic time* (period between organ extraction and transplant) as minimal as possible to increase the likelihood of successful transplantation. Patients on waiting lists need to be assessed carefully by specialists on a regular schedule and are required to be available on short notice, should an organ become available”. In this respect, there are linkages between the three models and poor performance in one stage is likely to affect the performance of the other stages.

However, despite these linkages, it is useful to analyze the results of each model separately because poor performance in each of these stages requires different improvement action plans. Poor performance in model 1 points to the need to increase resources, whilst poor performance in model 2 points towards the need to improve the activities related with harvesting and removal of organs. Finally, poor performance in model 3 points towards the need to improve the activities related with organ allocation, transplantation and patient follow up.

To measure each Brazilian state's performance, we have used the Efficiency Measurement System (EMS) software [45], and DEA models with output orientation. Having taken into account that the ODT process goals are to maximize the quantity and quality of the transplant services provided rather than to minimize the resources used in the process, the output-oriented models are the most appropriate models for this study. This is also in line with the studies by Siqueira and Araujo [9], Marinho and Araujo [21] and Costa et al. [36]. Regarding the scale assumption, CRS and VRS assumptions were used in the study since one of the objectives of the study is to assess the impact of scale in the provision of organ transplant services. Using both assumptions allows us to verify whether the states poor performance is related to management problems, scale problems or both. We also used weight restrictions related to some inputs and outputs, in order to guarantee reliable results, discrimination in the analysis and also to incorporate some production trade-offs between variables [46]. The incorporation of these weight restrictions ensured that no variable was assigned a null weight, meaning that no slacks are observed in the performance results of the three models. Please, refer to Table B in the Supplementary Material for more details about the weight restrictions incorporated in the models.

### Data and results

#### Data

The research sample consists of 17 Brazilian states plus the Federal District, in the five Brazilian regions that act in the process of organ donation and transplantation services linked to the Brazilian Unified Health System and the analysis period is from 2018 to 2020. The sample selection criterion was the participation of the Federative Unit in the ODT services and the complete availability of information. The states of Amapá, Roraima and Tocantins were excluded from the analysis because according to the Brazilian Transplant Registry (RBT) [19], they do not perform solid organ transplants. The states of Amazonas, Alagoas, Mato Grosso and Sergipe were excluded from the analysis because they did not have information available on transplant teams in some of the years analyzed. As indicated previously, transplant teams represent a fundamental resource of the ODT process. Excluding these states allowed us to present a balanced panel for all the years analyzed and perform a dynamic analysis to explore the impact of the COVID-19 pandemic. Acre and Rondônia were initially included in the analysis but they were later excluded as a result of being identified as outliers in some of the models. In order to identify the outliers we used the procedure proposed by Banker and Chang [47].

As discussed by Dyson et al. [48], when performing a comparison with DEA, units are assumed to be similar in several ways: firstly, units are assumed to undertake similar activities and produce comparable products and/or services; secondly, units are assumed to have access to a similar range of resources; and thirdly, units are assumed to operate in similar environments. The units compared in this study are homogeneous in terms of the activities developed because all 18 units perform solid organ harvesting and transplant activities. All units have access to a similar range of relevant resources (such as ICU beds, transplant beds and transplant teams). With regard to the environment in which the units operate, it is an objective of this study to compare the average performance levels of each region to verify if there are significant differences in performance between regions.

The data used in this study was collected from the website of the Department of Informatics of the Unified Health System (DATASUS) of the Ministry of Health of Brazil, on the basis of the System of Hospital Admissions of the SUS—SIH/SUS, and the National Registry of Health Establishments—CNES; and from the Brazilian Transplant Registry (RBT) available on the Brazilian Association of Organ Transplantation (ABTO) website.

#### Descriptive statistics of the data and performance scores

Table 1 shows the descriptive statistics of the variables used in the study using data from 2018 to 2020. As it can be observed, there seem to exist significant discrepancies in the data among the Brazilian states under analysis. For instance, looking at the number of patients on the transplant waiting list, we can see that while the average is 1473 patients, there are states with a much higher number of patients on wait. For example, while São Paulo had in 2020, 15587 patients on the waiting list, Goiás only had 126. Of course, the resources available in each state tend to reflect the needs of the respective populations served. Therefore, it is understandable that those states in the Southeast region that present the highest number of patients on the waiting list are also those that have the highest number of ICU beds, transplant beds and transplant teams (e.g. São Paulo, Rio de Janeiro e Minas Gerais). In fact, for most of the variables, the states of Minas Gerais, Paraná, Rio de Janeiro, Rio Grande do Sul and São Paulo tend to present numbers much higher than the average. On the opposite side, states in the North, Northeast and Midwest regions, tend to present the lowest values.

**Table 1:** Summary statistics for the variables used in the study

	Transplant waiting list	Nº of transplant beds	Nº of transplant teams	Nº of ICU beds	Nº of family consents	Nº of effective donors	Donors whose organs were transplanted	Transplant service expenses (*)	Nº of transplants performed	Nº of patient survivors
Average	1473.0	53.4	14.5	2701.7	441.5	192.9	163.5	23521.7	396.0	378.6
ST Dev	3107.0	61.2	18.1	3286.4	535.4	256.5	207.9	32210.6	535.5	511.1
Max	15587.0	259.3	81.0	17471.0	2478.0	1094.0	923.0	141025.4	2418.0	2301.0
Min	78.0	3.0	1.0	296.0	42.0	4.0	4.0	126.2	4.0	4.0
Skewness	3.7	2.1	2.4	2.7	2.7	2.4	2.4	2.4	2.5	2.5
Kurtosis	13.6	4.8	6.5	8.8	7.9	6.5	6.7	6.4	7.0	7.0

(*) Values in thousand Brazilian Reals

We also present results for the standard deviation, the maximum and the minimum values observed for each variable used in the study. These results show that the values observed in the states vary significantly around the mean (the standard deviations and the ranges are relatively high). Furthermore, in order to understand which type of statistical distribution best represents each variable, we also present the skewness and kurtosis. Skewness values are all larger than one, revealing data with positive skewness. Kurtosis values are also all larger than one, revealing leptokurtic distributions. These measures reveal that the size of the Brazilian states in terms of installed capacity and transplant activity varies considerably. For this reason, in order to distinguish between pure management problems and scale problems, we decided to run models with both Constant Returns to Scale (CRS) and Variable Returns to Scale (VRS) assumptions.^2^

Regarding the impact of COVID-19 on the ODT process in Brazil, when we compare the values of the variables in 2018 (pre-pandemic) with those in 2020 (pandemic period), we can observe that although the number of transplant teams and the number of family consents for ODT increased slightly, in 1.9% and 4.6%, respectively, the number of effective donors and transplants performed suffered a reduction of 5.2% and 23.9%, respectively. The transplants from living donors experienced the biggest drop, as they represent elective surgeries which were suspended for varying periods in most states [19]. Although these figures suggest a detrimental effect, the impact of the COVID-19 pandemic on the performance of the ODT process is discussed in more detail later. In what follows, we present the summary statistics of the results obtained by the three models used in the study (Table 2).

**Table 2:** Summary statistics of the results

		2018			2019			2020		
		Technical Performance	Pure Technical Performance	Scale Performance	Technical Performance	Pure Technical Performance	Scale Performance	Technical Performance	Pure Technical Performance	Scale Performance
Model 1 – Equity in the Allocation of Resources for Transplantation	Average	59%	80%	76%	55%	77%	73%	45%	77%	59%
	St Deviation	23%	24%	20%	26%	27%	24%	21%	24%	22%
	Maximum	100%	100%	100%	100%	100%	100%	100%	100%	100%
	Minimum	17%	23%	21%	14%	23%	19%	9%	22%	13%
Model 2 – Organ Donation and Harvest	Average	65%	84%	79%	61%	81%	76%	63%	75%	83%
	St Deviation	24%	22%	23%	26%	21%	23%	27%	26%	18%
	Maximum	100%	100%	100%	100%	100%	100%	100%	100%	100%
	Minimum	13%	21%	24%	12%	30%	12%	12%	14%	40%
Model 3 – Organ Transplantation	Average	74%	91%	81%	83%	93%	89%	75%	91%	82%
	St Deviation	18%	9%	16%	16%	8%	13%	16%	9%	13%
	Maximum	100%	100%	100%	100%	100%	100%	100%	100%	100%
	Minimum	47%	75%	56%	52%	75%	63%	52%	68%	59%

Regarding the overall average score for the three models, model 3 shows the best average and the smallest variation among the results, and model 1, has the worst, resulting in a large standard deviation among the results. This indicates that the discrepancies among Brazilian states are much smaller in terms of organ transplantation than in terms of equity in the distribution of the resources for organ transplantation. The results suggest, however, that there is considerable scope for improvement in each of the three dimensions. In fact, the results of models 2 and 3 indicate that the average performance scores obtained by the states range from moderate to weak. In which regards model 1, the results indicate a relatively poorer performance for the three-year period. Therefore, while the procedures related with organ donation and harvest and with organ transplantation can still be improved, it is the equity of resource allocation that seems to require the most attention, as this part of the ODT process is the one that reveals the worst results. This is a relevant finding because an inequitable distribution of resources for organ transplantation has a direct impact on organ harvest and transplantation processes.

Regarding the three models used, we can also observe that there is an increase in the average scores and in the number of states considered best practices in the VRS models when compared to the CRS models. This indicates that some states, such as São Paulo, for example, might have a good management performance, but do not have the right scale of operations in which regards the ODT process. The fact that large differences between the CRS and VRS scores are observed for some states is also an important finding as it confirms the effect of scale on the performance of the states. There are, however, a number of states (e.g. Espirito Santo, Rio de Janeiro) which present poor performance scores under both assumptions, indicating that they suffer from both managerial and scale problems.

#### Geographical distribution of the performance scores

Considering that Brazil is a country with continental dimensions, which is divided into five geographic regions and 26 states plus the federal district, with different demographic, climatic, economic, social, and health conditions [50], this section aims to analyze the geographical distribution of the performance scores. In order to do so, we take the results regarding the comparison of the 18 DMUs between themselves and analyze the average performance scores by geographic region (please, refer to Table 3).

**Table 3:** Average performance scores by geographic region

		2018			2019			2020		
		Technical Performance	Pure Technical Performance	Scale Performance	Technical Performance	Pure Technical Performance	Scale Performance	Technical Performance	Pure Technical Performance	Scale Performance
Model 1 – Equity in the Allocation of Resources for Transplantation	North Region	66%	74%	89%	60%	61%	100%	23%	39%	60%
	Northeast Region	59%	73%	82%	60%	79%	76%	45%	72%	66%
	Midwest Region	65%	87%	78%	46%	56%	86%	72%	87%	83%
	South Region	80%	94%	85%	73%	93%	78%	50%	90%	55%
	Southeast Region	37%	77%	55%	36%	81%	48%	24%	78%	33%
Model 2 – Organ Donation and Harvest	North Region	55%	100%	55%	42%	100%	42%	19%	47%	40%
	Northeast Region	58%	85%	69%	58%	81%	70%	59%	71%	83%
	Midwest Region	59%	74%	83%	47%	57%	85%	55%	69%	85%
	South Region	86%	92%	94%	82%	91%	91%	86%	92%	94%
	Southeast Region	68%	79%	87%	67%	85%	79%	70%	82%	86%
Model 3 – Organ Transplantation	North Region	100%	100%	100%	100%	100%	100%	100%	100%	100%
	Northeast Region	82%	93%	87%	86%	93%	92%	77%	90%	85%
	Midwest Region	77%	86%	90%	86%	90%	96%	74%	89%	84%
	South Region	55%	85%	64%	67%	87%	76%	62%	88%	70%
	Southeast Region	66%	93%	71%	84%	97%	86%	75%	95%	79%

Before the results are discussed, it is important to bear in mind that although the South and Southeastern regions cover only about 20% of Brazil’s territory, they concentrate 57% of the Brazilian population, 73% of the Gross Domestic Product (GDP) and most professionals affiliated to the Brazilian Transplantation Society. In contrast, the North region, which covers the largest part of the Brazilian territory, has the lowest population density and is the second poorest region [17, 50]. It is no surprise, therefore, that the results of model 1 indicate a higher equity in the allocation of resources for transplantation in the South Region than in the North, reflecting a much higher availability of transplant beds and transplant teams in relation to the patients on the waiting list in the South than in the North. In addition, it is important to consider that regions with greater population density and easier access tend to allow for a higher number of transplant services and organ donation and harvesting services, as the patients in these regions need to travel less for pre-and post-transplant appointments. Furthermore, in these regions, there is a greater number of nearby donors and a greater number of health professionals trained in transplantation [17, 44, 51]. This probably justifies why, when it comes to the organ donation and harvest process, captured by model 2, states in the South and Southeast regions tend to perform better. In fact, the states in these regions have one of the lowest rates of family refusal to donate organs and one of the highest rates of conversion of potential donors into real donors among the Brazilian states [19]. As can be observed from Table 3, our results seem to confirm this, as the states that achieve higher performance scores in model 2 are those located in the South and Southeast regions. Interestingly, however, our results do not confirm the commonly held view that these regions also have better performance in organ transplantation [9]. This is probably due to the fact that although the states in the South and Southeast regions perform a much higher number of transplants, the expenses associated with the transplant services and the resources allocated to transplantation are much higher than those in other regions. As the results of Table 3 suggest, the states in the South and Southeast regions are the ones that display more scale problems when it comes to the organ transplantation process, captured by model 3.

Although these results offer valuable insights, the variation observed in the performance scores of the Brazilian states across regions does not allow us to establish a clear performance profile directly related to each of the 5 Brazilian regions. For example, in which regards model 1, our results show that Santa Catarina is the only state that reaches the score of 100% both in the model with a CRS assumption and a VRS assumption in 2018. In 2019, the number of states reaching a 100% score increases to three: Paraíba, Piauí and Santa Catarina, while in 2020 only the state of Goiás reaches the maximum performance. In the VRS model, Minas Gerais, Paraná and São Paulo were among the best performers during the three years under analysis. These results, together with the fact that these states reach considerably lower scores when the CRS assumption is used, suggest that while the management of the ODT process seems to be appropriate, there are substantial problems related to the scale of operations. There are a number of states, however, that in addition to displaying scale problems also display management issues. This is the case of states such as Bahia and Rio Grande do Sul. Interestingly, with the exception of the state of Goiás, the results suggest that all other Brazilian states would have benefited from a reduction in the scale of operations in 2020 (pandemic year) indicating that the capacity installed was excessive in comparison to the services being provided, probably as a result of the impact of COVID-19 on organ donation and transplantation, an issue which we will explore in further detail in the next section. It is important to emphasize, however, that despite the existence of inequality in physical and human resources as well as logistical limitations among the states for the provision of organ donation and transplantation services, based on the analysis developed, it is not possible to identify clear geographical patterns related to scale performance for the Brazilian regions.

With regard to organ donation and harvest (model 2), Santa Catarina is the only fully efficient state under the CRS and VRS assumptions in the three-year period under analysis. The state of Ceará also presents a very solid performance in terms of the organ donation and harvest process, obtaining a performance score of 100% in 2019 and 2020, and higher than 95% in 2018. Both states can represent important benchmarks for other states as considering the installed capacity in terms of ICU beds, transplant teams and family consents for organ donation, these are the states that are better converting their capacity into effective donors and into donors whose organs are transplanted. Ceará, for example, was referred to as a benchmark for the highest number of states including Pernambuco, Paraíba, Goiás and Minas Gerais. Therefore, Ceará can be used as a benchmark not only for states in other regions but also for states within its own region (e.g. Paraíba and Pernambuco). Because these neighboring states share socio and economic commonalities, this suggests that important learning networks can be formed as similar strategies can be applied. From 2018 to 2020, with the CRS assumption, less than a third of the states achieved scores above 80%, however, this number increases with the VRS assumption where Paraná, Piauí, Santa Catarina and São Paulo reach a score of 100%. However, we can see that states in the North and Northeast regions tend to present lower scores in the CRS model, which leads us to consider that much of their underperformance is related to scale. The states in these two regions seem to be also the ones that have suffered most as a consequence of the SARS-CoV-2 when it comes to the organ donation and harvest process as the performance of states like Pará (North region), Maranhão and Rio Grande do Norte (both from the Northeast region) are the ones that experienced the largest deterioration in their performances from 2018 to 2020. Interestingly, although these three states reached a low performance score in 2018 under the CRS assumption, they all reached a score of 100% in the VRS model. In 2020, their performances under both assumptions deteriorated significantly, suggesting that in addition to the scale problems, these states seem to have experienced the most difficulties in adapting to the new circumstances brought up by the COVID-19 pandemic, which generated a sudden change in hospital logistics, access to ICU beds, and the addition of new protocols for performing organ donation and transplantation. However, like in model 1, the variation observed in the average of the performance scores of the Brazilian states does not allow us to establish a clear performance profile directly related to each of the Brazilian regions.

Regarding the performance of the states in terms of the organ transplantation process (model 3), our results show that the state of Pará achieved maximum performance, both under the CRS and VRS assumptions, for the years under analysis, while Paraíba achieved the maximum score in 2018, Maranhão and Minas Gerais in 2019 and Rio Grande do Norte in 2020. Considering that each of these states underperformed in terms of the organ donation and harvest process, this clearly indicates that there are opportunities for reciprocal learning between states. For example, the state of Pará can enhance its performance in terms of this part of the ODT process by following the practices of the state of Santa Catarina, and Santa Catarina can improve its performance in terms of the transplantation process following the practices of Pará. However, we would like to highlight that some of the states that achieve maximum performance in terms of organ donation and harvest also achieve maximum performance in terms of organ transplantation, in particular when we consider the VRS assumption (e.g. Piauí and São Paulo). As model 2 focuses more on evaluating the states regarding organ donation and harvesting and model 3 has a greater focus on the number of transplants performed and patients that survive, the results of both models may not necessarily be consistent. Therefore, it is important that health managers and policymakers seek strategies and corrective actions directed to each of these parts of the ODT process, which can be substantially different. Although both models are directly related to the ultimate goal of offering organ transplants to patients in need, they focus on different parts of the process. Also, when it comes to organ transplantation, the scale of operations seems to play an important role in explaining the poor performance of some states (e.g. São Paulo), as these states have a low score in the CRS model but reach a 100% score in the VRS model.

#### Assessing the impact of COVID-19 on the performance of the states

Although the analysis performed in the previous sections seems to suggest that the COVID-19 pandemic might have had an important effect on the performance of the ODT process carried out by the different states, it is important to bear in mind that the results obtained for each year are determined by the frontier of the respective year. Therefore, the results do not allow comparisons between frontiers and performance scores obtained in different years to take place. In order to address this limitation, we performed a dynamic analysis using the Malmquist Productivity Index, which allows comparisons of data from different years.^3^ Considering that there is considerable scale heterogeneity between the Brazilian states, following the suggestion of Zarrin et al. [53], we undertook cluster analysis to form groups that are more homogeneous in terms of size. Two clusters were formed, one with 11 DMUs of relatively small size in terms of their capacity to perform organ transplants and another cluster with seven DMUs of relatively larger size in terms of installed capacity.^4^ The MPI analyses were undertaken with separate frontiers for these two clusters. Table 4 contrasts the geometric mean values for the MPI and its components—catch-up effect and frontier shift effect—for the three years under analysis, for each cluster, in each model used.

**Table 4:** The Malmquist Index and its Components by Cluster

		2018–2019			2019–2020			2018–2020		
		C	F	MPI	C	F	MPI	C	F	MPI
Model 1 – Equity in the Allocation of Resources for Transplantation	Cluster 1 (Federal District and states with relatively low transplant capacity)	0.94	1.09	1.03	0.87	1.20	1.04	0.82	1.39	1.14
	Cluster 2 (States with large transplant capacity)	1.11	0.81	0.89	0.92	1.02	0.94	1.02	0.82	0.84
Model 2 – Organ Donation and Harvest	Cluster 1 (Federal District and states with relatively low transplant capacity)	0.93	1.02	0.95	0.99	0.84	0.84	0.92	0.85	0.79
	Cluster 2 (States with large transplant capacity)	0.91	1.13	1.03	1.07	0.79	0.84	0.97	0.89	0.87
Model 3 – Organ Transplantation	Cluster 1 (Federal District and states with relatively low transplant capacity)	1.09	0.89	0.97	0.91	0.94	0.85	0.98	0.86	0.84
	Cluster 2 (States with large transplant capacity)	1.03	0.95	0.97	0.97	0.94	0.92	1.00	0.89	0.89

Note: C—Catch-up Effect (Efficiency change); F—Frontier Shift (Technological Change); MPI—Malmquist Productivity Index

Although Table 4 reports changes from 2018 to 2019 and from 2019 to 2020, our analysis focuses mostly on the changes observed from 2018 to 2020 as these two years allow us to contrast a pre-pandemic period with a period marked by the pandemic. In which regards the results obtained for cluster 1 related to the equity in the allocation of resources for transplantation (Model 1), these need to be interpreted with caution as the frontiers in 2018 and 2020 are defined by a single state – Santa Catarina in 2018 and Goiás in 2020. Therefore, although the results of the MPI from 2018 to 2020 seem to suggest a significant and favorable frontier shift in terms of the equity in the allocation of resources (component F) in cluster 1, these results essentially reflect the fact that Goiás in 2020 had a much higher level of resources available to perform transplants in comparison to the population in the waiting list than those offered by Santa Catarina in 2018. This effect is justified in part by the drop in 41.7% of the patients on the waiting list in Goiás from 2018 to 2020, suggesting that this state in 2020 had the largest amount of resources available in relation to the population needs. In this respect, the average MPI for all states belonging to cluster 1, including the Federal District, which combines both changes in the positioning of the frontier and in the distances of the states to the frontier, shows a value higher than one, indicating a 14% improvement in equity. This improvement occurred despite the fact that, on average, cluster 1 states in 2020 were further away from the frontier than they were in 2018. This is captured by the catch up effect (component C), which is smaller than one in this cluster.

A different evolution in terms of equity is observed in cluster 2. In this case, the best practice frontier of model 1 in 2020 is at a lower level than that observed in 2018 (component F). The justification for this lies in the fact that, in cluster 2, on average, the size of the waiting lists increased 19% from 2018 to 2020, but the resources allocated to these states did not increase. Therefore, despite that, on average, states from cluster 2 were in 2020 slightly closer to the best practice frontier than in 2018, the fact that the frontier regressed significantly, led to a decrease in terms of equity.

The results of the other two models are also important to assess the impact of COVID-19 in this sector. As the results suggest, in both models 2 and 3, from 2018 to 2020 there was a significant decrease in the average productivity of the states belonging to both clusters. This decrease is mainly explained by a regression in the best practice frontier in 2020 compared to 2018, which, at least in part, is likely to be due to the COVID-19 outbreak. In model 2, although capacity seems to have increased in some states, this growth was not matched by a similar increase in the number of effective donors and donors whose organs were transplanted. In regard to the productivity of the states in terms of the variables captured by model 3, the average performance of the states is very similar to the one observed in model 2. Interestingly, the results show that either at the level of the organ donation and harvest process or at the level of the organ transplantation process there was a clear decline in both clusters from 2018 (pre-pandemic) to 2020 (pandemic period).

**Fig. 3:**
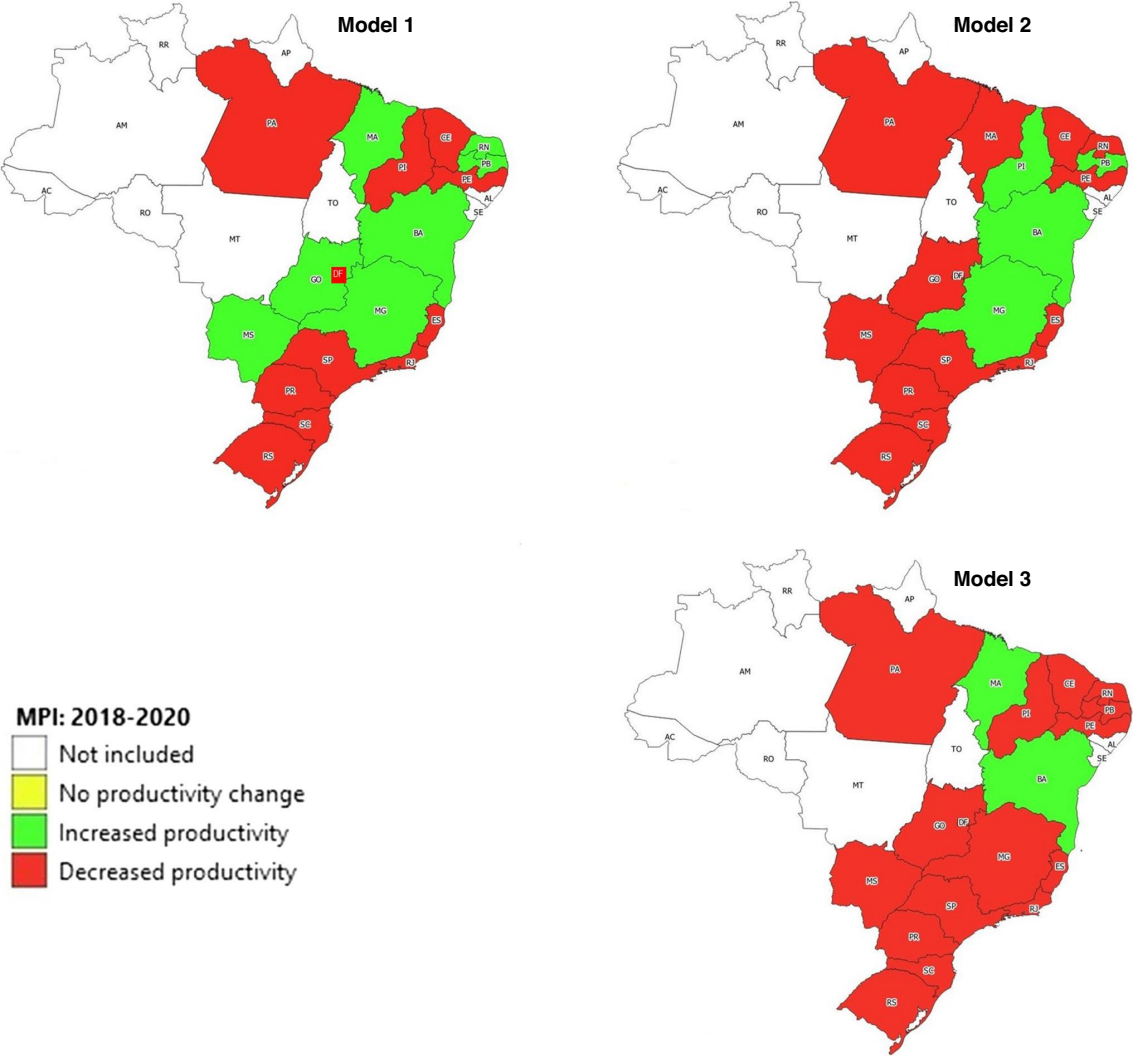
MPI results for the 2018–2020 period by State plus the Federal District. Note: North Region—AM = Amazonas, AP = Amapá, RR = Roraima, PA = Pará, AC = Acre, RO = Rondônia, TO = Tocantins. Northeast Region—MA = Maranhão, PI = Piauí, CE = Ceará, RN = Rio Grande do Norte, PB = Paraíba, PE = Pernambuco, AL = Alagoas, SE = Sergipe, BA = Bahia. Midwest Region—MT = Mato Grosso, MS = Mato Grosso do Sul, DF = Federal District, GO = Goiás. Southeast Region—MG = Minas Gerais, ES = Espírito Santo, RJ = Rio de Janeiro, SP = São Paulo. South Region—PR = Paraná, SC = Santa Catarina, RS = Rio Grande do Sul

The results of our analysis show, however, that the pandemic did not affect all the states in the same way. This can be confirmed through the analysis of Fig. 3.

As illustrated by Fig. 3, the procedures of organ transplantation seem to have been the ones most affected by the COVID-19 pandemic as it is in this part of the ODT process that we observe a higher number of states with a MPI score below 1, when we compare the activity of 2018 with that carried out in 2020. In fact, only Bahia and Maranhão, two states with small transplant capacity in the Northeast region, show gains in productivity due exclusively to the fact that with the decline in the frontier, they were able to get closer to it. Overall, however, there was a productivity decrease in this part of the ODT process of around 16%. The states that seem to have experienced more difficulties in handling the pandemic are Paraíba, Pernambuco and Goiás. For these states, we observe not only a decline in the frontier but also an increase in the distance of these states to the respective frontier.

The COVID-19 pandemic seems to have had also a detrimental influence in terms of the organ donation and harvest process, as overall, the MPI shows a decrease even larger than that observed for organ transplantation. Again, this originated mostly from the decline in the frontier, indicating that the states faced more difficulties in 2020 than in 2018 in converting family consents into effective donors and donors whose organs were transplanted, despite their installed capacity in terms of ICU beds and transplant teams. In this part of the ODT process, the states that displayed more difficulties were Pará, Maranhão and Rio Grande do Norte, three states with small installed transplant capacity, all belonging to the North or Northeast regions.

In a nutshell, our results show that from 2018 to 2020 most Brazilian states and regions experienced a significant drop in productivity in terms of the organ donation and transplantation process as well as in terms of the equity in the allocation of resources for transplantation, with the exception of the Midwest and Northeast region in model 1. Because the COVID-19 pandemic was a major structural factor taking place in between the two periods analyzed, it is reasonable to consider that the pandemic was the major driving force behind the observed decrease in productivity. This conclusion is supported not only by the results of our analysis but also by recent literature reflecting on the impact of the pandemic on transplant services. As discussed by Georgiades et al. [54], Loupy et al. [55] and Cholankeril et al. [56], a drastic reduction in solid transplant activity was observed worldwide due to the pandemic. Several reasons explain this reduction in transplant activities. Firstly, the need to allocate as many ICU beds as possible for COVID-19 patients, implied a lower availability of ICU beds for transplant services [57]. Secondly, the reduced availability of staff during the pandemic (due to the need to treat COVID-19 patients and due to high infection rates of the professionals) also implied that fewer human resources were available to perform transplants [4]. Thirdly, the reduced number of potential donors, related with the reduced number of neuro critical patients’ admissions and also with the risk regarding the donor’s being infected with SARS-CoV-2 meant that less organs were available to be transplanted [58]. Fourthly, organ procurement and organ transplant procedures involve a high risk of SARS-CoV-2 transmission, leading to the development of strict guidelines, which negatively impacted on the level of transplant activities [4].

### The practical and policy implications of the results

The results shown previously lead us to the conclusion that independently of the situation caused by the COVID-19 pandemic, there is considerable potential for Brazilian states to improve their performance and achieve better outputs related to the entire process of organ donation and transplantation. This conclusion applies to all parts of the process ranging from the equity in the allocation of resources to the procedure of the transplant itself. In particular, the results indicate that there is a gap between the services some states can achieve and their current performance. Those states, which present potential for performance improvement in some of the dimensions, can benefit from learning from states that score 100% in the models studied. Furthermore, the information obtained from the results can be a beneficial source for policymakers, program planners and health managers. Through the identification of best practices, the study shows the states whose actions related to the ODT process should be studied with the intention of being replicated by states that have not yet reached high levels of performance.

As this study worked with different models, ranging from models able to assess the equity in the allocation of resources for transplantation (model 1) to models focused on the organ donation and harvest process (model 2) and organ transplantation services (model 3), the results of our analysis indicate that some of the states that perform very well in a part of the process, do not necessarily perform well in other parts, suggesting opportunities for reciprocal learning. In fact, no Brazilian state has achieved a score of 100% for the three models, from 2018 to 2020, in CRS and VRS assumptions. For model 2, Santa Catarina was the only state that achieved a 100% score during the full period analyzed in both scale assumptions and Pará for model 3. In relation to model 1, no state scored 100% in all years analyzed. Santa Catarina scored 100% in 2018 and 2019, while Goiás was the only state to reach a score of 100% in 2020.

If we analyze the performance only under the VRS assumption, São Paulo achieves a score of 100% in the three models of the period studied, which means that São Paulo does not present management problems, although scale problems were identified. However, given the results obtained, we cannot establish a single state as a reference to be studied for the different parts of the ODT process. Regarding the equity in the allocation of resources for transplantation (model 1), Santa Catarina is one of the states that presents the best results for this model in the period before the COVID-19 pandemic. For organ donation and harvest (model 2), Santa Catarina and Ceará appear as important benchmarks for a considerable number of states, which makes them states of interest related to best practices. These two states have a high number of family consents for organ donation, and more than 50 per cent of the number of family consents for organ donation become effective donors, which can be considered important strengths of these states in the organ donation and harvest process. In relation to organ transplantation (model 3), Pará stands out, serving as a benchmark for a large number of states. However, in addition to the states mentioned above, which can be used as benchmarks, it may be interesting that states with potential to improve their performance try to learn from their respective benchmarks within the same region since they share very similar characteristics making it easier to develop suitable improvement plans.

Our results also offer important insights regarding the effect of COVID-19 on the ODT process and how each state handled the challenges posed by the pandemic. Due to the fact that our study used the years from 2018 to 2020 as the time frame for analysis, it enabled us to measure the changes in productivity before the COVID-19 outbreak as well as the impact of the pandemic on each part of the ODT process and on each state. Regarding the equity in the allocation of resources for transplantation, the results show a slight improvement in the average performance of the states with small capacity plus the Federal District due mostly to the outwards movement of the respective frontier. This movement is explained by a significant drop in the number of patients on the waiting list in the state of Goiás in 2020. However, a different situation is observed in states with large transplant capacity, which experienced a deterioration in equity because resources did not increase to accommodate for the increase observed in the size of the waiting lists. When we analyze the results from models 2 and 3, related to the organ donation, harvest and transplantation processes, we observe a very significant deterioration in the overall performance of the states explained mostly by a decline in the respective frontier. These results indicate that despite the installed capacity not suffering significant changes from 2018 to 2020, in this latter year, the states offered a much lower level of services compared to those offered in 2018. These results are suggestive of the COVID-19 pandemic having a considerable detrimental effect in relation to transplant surgeries in Brazil. However, considering that some states seem to have fared better than others in handling the pandemic, important policy lessons might also be derived from studying these states in detail.

## Conclusion

Although organ transplantation is one of the best treatment options for a large and growing number of medical conditions, the results of this study show that the pandemic caused by the novel coronavirus SARS-CoV-2 might have detrimentally impacted the provision of transplant services to many patients in need. In fact, the results of our analysis seem to suggest that the pandemic exacerbated some of the challenges already experienced by the transplant providers prior to the beginning of the pandemic. Taking into account the case of Brazil, it is estimated that the annual need for solid transplants—kidney, liver, heart and lung—is threefold the number of transplants performed. Only in 2020, 12757 new patients joined the waiting list [19]. Considering the high number of patients who need organ transplants and the financial impact this procedure has on public coffers, it is imperative to ensure strategies to increase and improve the quality and access to organ transplant services across the country, even during pandemic periods, as well as to ensure that resources are used as efficiently as possible.

In the literature, there have been important contributions—albeit still limited—exploring the use of DEA to assess the performance of the organ donation-transplantation process. However, a number of these studies focus only on kidney transplantation, mainly using the number of transplants performed as an output. In this paper, we have explored the potential of using DEA in order to complement the existing literature in the organ transplant area. For this purpose, we have proposed three complementary models to assess three performance dimensions and to compare the performance of Brazilian states in promoting solid organ donation and transplantation, using data from 2018 to 2020. The impact of COVID-19 on the provision of ODT services was also assessed.

Despite the exploratory nature of this study, there are some important findings that can be taken from this research. A wide variation in terms of performance both prior and during the pandemic was found among the states and models studied, indicating that some states have achieved considerably better results in the allocation of resources for organ transplantation than others, even when faced with the challenges posed by the novel coronavirus SARS-CoV-2. Consequently, there is great potential for improvement for all Brazilian states and regions, since all states have room for improvement in at least one of the proposed models. In addition, since these models assess the ODT process as a whole, it is of paramount importance to achieve good results in the three models. On top of that, a large number of states have problems related to the scale of operation, as they get better results in the VRS model. This must be taken into account, in order to operate on a scale that allows the needs of the population to be met without wasting resources. Based on the fact that our study, in addition to the identification of the performance of Brazilian states, allows identification of learning peers for each underperforming state, along with targets for performance improvement, we believe it can be crucial to support further assessments in this area. In particular, it can assist studies aimed at identifying the root causes of poor performance, examining the best performers' practices and assisting underperforming states to develop strategies for improving the ODT process.

The formative implementation of DEA in the ODT context, with the use of data collected from a reliable and standardized source for more than a period, also allows dynamic assessments to analyze possible changes in the performance of states over time. In addition, it allows obtaining consistent and robust results. However, as the data used in this study were collected by each Brazilian state, there may be some variations in this process. For example, on the waiting list for organ transplants, some states do not report having any patients on this list, as is the case of the states of Amazonas and Tocantins. Soares et al. [59] believe that this is much more related to the fact that there are no authorized transplant services in those states than the fact that there are no patients with a clinical indication for transplant. Although those states are not analyzed in our study, it raises a concern that there may be an underreporting of health needs for other Brazilian states due to lower concentrations of doctors and transplant services in some regions. Moreover, as discussed before, caution needs to be exercised in interpreting the results due to some limitations in the access to data. In particular, we highlight that no information was available on the types of transplant beds, meaning that it was not possible to distinguish between beds for solid and non-solid organs. No information was also available on the number of hours worked by each team or on the number of recipients who received more than one transplant. In this respect, to promote a fairer assessment, it is advised that this type of information is incorporated into future studies whenever it becomes available. Furthermore, future studies may want to explore the use of alternative models to evaluate the performance of organ transplantation systems, such as non-radial models and network DEA models. In particular, slack-based models can be useful as they allow performance assessment without having to deal with the complexity involved in defining appropriate weight restrictions.

In conclusion, the results found in this study point to the need for integration among the different steps necessary to perform organ transplants in order to achieve better outputs and a more adequate use of public spending. The proper integration of the ODT process and the efficient use of resources are of paramount importance in a country like Brazil due to the underfunding of public services, in general. Low levels of performance in the studied models lead, not only to waste of public resources but also to less availability of transplant services for the population who need them, especially in a country with high social disparities and where over 90% of organ transplants are financed by the government. Due to scarce resources and increasing needs, the health sector is particularly dependent on the efficient use of resources to achieve the results necessary to meet population needs.

### Electronic supplementary material

Below is the link to the electronic supplementary material.

Supplementary file1 (DOCX 23 KB)

## Data Availability

Data will be made available on request.

## Appendix

1. **An Introduction to Data envelopment Analysis**

There are a number of considerations involved in developing a DEA model. In particular, it is important to (1) select the decision-making units for analysis; (2) define the models by selecting the input and output variables; (3) select the model orientation; and (4) select the type of returns to scale assumption. It is of paramount importance to perform these steps properly, as each of them has the power to affect the insights gained through the analysis [60].

A DEA model can be input-oriented, output-oriented or non-oriented. An input-oriented model aims to estimate the minimum possible level of resources used to produce a certain amount of outputs, while an output-oriented model aims to determine the maximum level of outputs produced with a certain quantity of inputs [31, 61, 62]. The non-oriented model combines both orientations in a single model (Cooper et al., [63]). Basically, the choice of the type of model is determined by the controllability over the variables [29].

Regarding the returns to scale assumption, the two basic DEA models are the CCR and the BCC. “Despite countless extensions, these early models are still the most frequently utilized models” (Kohl et al. [30: 253]). The models with “constant returns to scale (CCR) assume that changes in the inputs result in proportional changes in the outputs, while variable returns to scale (BCC) assume that an increase (or decrease) in the inputs does not necessarily lead to proportional changes in the outputs” (Kalinichenko et al., [64: 1088]). The efficiency estimate obtained by the CCR model is called technical efficiency, in which DMUs are compared to each other, regardless of the size at which each one operates [32, 62, 65]. Conversely, in the BCC model, the DMUs are compared only with DMUs that operate on a scale similar to their own. Consequently, the size of the operations is relevant. The efficiency obtained with the BCC model is named pure technical efficiency [32, 62, 65]. The BCC model makes it possible to assess technical inefficiency from two perspectives, which are scale inefficiency and management inefficiency [32]. According to Cantor and Poh [60], most studies in the health literature use both assumptions for analysis.

The model that allows the calculation of the relative efficiency score of each DMU assuming a CRS assumption is written in (1). Consider a sample of *n* DMUs to be compared, using *m* inputs to produce *s* outputs. Assume that DMU_*j*_ is under evaluation and that *x*_*ij*_* and* y_*rj*_ are the levels of the *i*th input and *r*th output observed for DMU_*j*_. The solution of model (1), proposed by Charnes et al. [27: 432], allows the calculation of the efficiency score of a certain DMU_0_ $\left(z_0=1/g_0\right)$ and the optimum weights of each input $\left(w_i\right)$ and each output $\left(\mu_r\right)$:

1 $${\mathrm{min}g}_{0}=\sum\nolimits_{i=1}^{m}{w}_{i}{x}_{i0}$$

subject to:

$$\begin{array}{l}\begin{array}{ll}-\sum\limits_{r=1}^{s}{\mu }_{r}{y}_{rj}+\sum\limits_{i=1}^{m}{w}_{i}{x}_{ij}\ge 0;& j=1, \dots , n.\end{array}\\ \sum\limits_{r=1}^{s}{\mu }_{r}{y}_{r0}=1\\ \begin{array}{ccc}{\mu }_{r}, {w}_{i}\ge 0;& r=1, \dots , s;& i=1,\dots ,m\end{array}\end{array}$$

Furthermore, the solution to the dual of model (1), as proposed by Charnes et al. [27: 431–432], allows the identification of the benchmarks and targets for improvement, as shown in model (2):

2 $${\mathrm{max}z}_{0}$$

subject to:

$$\begin{array}{l}\begin{array}{ll}-\sum\limits_{j=1}^{n}{y}_{rj}{\lambda }_{j}+{y}_{r0}{z}_{o}\le 0;& r=1, \dots , s\end{array}\\ \begin{array}{ll}\sum\limits_{j=1}^{n}{x}_{ij}{\lambda }_{j}\le {x}_{i0}& i=1, \dots , m\end{array}\\ \begin{array}{ll}{\lambda }_{j}\ge 0& j=1,\dots ,n.\end{array}\end{array}$$

If we subtract an intercept term $({w}_{0})$ to the objective function and also to the left-hand side of the first restriction in model (1) we obtain the BCC model (variable returns to scale), proposed by Banker et al. [28]. This intercept term generates an additional restriction in model (2), imposing that the sum of the intensity variables $({\lambda }_{j})$ is equal to one.

The CCR and the BCC models, as initially proposed, only establish that the weights to be attributed to the inputs and to the outputs must be positive. However, this can lead to evaluations based on a weight structure that is not consistent with prior knowledge about the relationship between inputs and outputs. For example, some important inputs or outputs may end up with a very small weight. For this reason, some authors have proposed the introduction of weight restrictions in DEA models (see, for example, Dyson and Thanassoulis [66], Podinovski [46]). The use of weight restrictions allows the introduction of information about importance judgements or production trade-offs regarding the inputs and/or outputs.

2. **An Introduction to the Malmquist Index**

Consider that, under the CRS assumption, ${\delta }^{{t}_{2}}(({x}_{0},{y}_{0}{)}^{{t}_{1}}$ with *t*_*1*_ = 1,2 and *t*_*2*_ = 1,2 indicates the efficiency rate of DMU_0_ operating in time period t_1_, evaluated with regards to the frontier of period t_2_. According to Tone [67], we can use the following expressions to calculate the ‘catch-up index’ (C) and the ‘frontier-shift index’ (F):

3 $$C=\frac{{\delta }^{2}(({x}_{0},{y}_{0}{)}^{2}}{{\delta }^{1}(({x}_{0},{y}_{0}{)}^{1}}$$

4 $$F={\left[\frac{{\delta }^{1}(({x}_{0},{y}_{0}{)}^{1}}{{\delta }^{2}(({x}_{0},{y}_{0}{)}^{1}}\times \frac{{\delta }^{1}(({x}_{0},{y}_{0}{)}^{2}}{{\delta }^{2}(({x}_{0},{y}_{0}{)}^{2}}\right]}^{1/2}$$

The expression for the MPI can be obtained by multiplying both effects:

5 $$MPI={\left[\frac{{\delta }^{1}(({x}_{0},{y}_{0}{)}^{2}}{{\delta }^{1}(({x}_{0},{y}_{0}{)}^{1}}\times \frac{{\delta }^{2}(({x}_{0},{y}_{0}{)}^{2}}{{\delta }^{2}(({x}_{0},{y}_{0}{)}^{1}}\right]}^{1/2}$$

When calculating the intertemporal efficiency scores ${\delta }^{{t}_{2}}(({x}_{0},{y}_{0}{)}^{{t}_{1}}$ and ${\delta }^{{t}_{1}}(({x}_{0},{y}_{0}{)}^{{t}_{2}}$, the exclusive scheme tends to be used (that is, the DMU under evaluation is excluded from the evaluation group, which is equivalent to super-efficiency evaluation developed by Andersen and Petersen [68]). In this respect, the efficiency score, if it exists, can be greater than 1.

A MPI greater than one suggests progress in productivity, less than one suggests a decrease in productivity, while equal to one means maintenance of the status quo [33]. The MPI allows us to determine whether changes in productivity are driven by frontier shifts or by technical efficiency changes, known as the catch-up effect [25]. The catch-up effect indicates whether a particular DMU is moving closer or further away from its corresponding efficiency frontier through increases or decreases in its technical efficiency from one period to another while the frontier-shift effect shows changes in the frontier itself which occur when there is a change in the industry to which this unit belongs [9, 25, 33]. When the frontier improves (deteriorates) considerably in the period evaluated, it may indicate that there has been significant technological progress (regress) or legal changes in the sector. Changes in the frontier can also reflect the impact of environmental factors such as the COVID-19 in the health sector.

The MPI can also be calculated using a VRS assumption. However, as emphasized by Thanassoulis [52], MPI should be calculated using a CRS assumption.

## Footnotes

1 For the variable number of transplant beds, the data are available monthly, so the annual average was calculated. The number of medical teams related to solid organ transplantation is derived from the sum of teams for each of the types of solid organ transplantation. The number of family consents is calculated from the difference between potential donors and family refusals to donate organs and tissues. The number of patient survivors is calculated from the difference between transplants performed and hospital deaths. Effective donors represent “the number of brain death notifications minus the number of potential donors lost because of factors such as medical contraindications, family refusal, and maintenance failure” (Siqueira and Araujo [9: 7]).

2 Following the proposal of Banker [49], we have tested the null hypothesis of constant returns to scale using the F test. Both assuming an Exponential and a Half-Normal distribution for the inefficiency variable, the null hypothesis of CRS was rejected for the three models and the three years (with 5% probability in all models and all years, apart from model 2 in 2020 – in this case, the null hypothesis was rejected with 10% probability). For that reason, we consider it to be relevant to present both CRS and VRS results.

3 When undertaking the analysis with the Malmquist index, we used the CRS assumption because, as discussed by Thanassoulis [52], the CRS assumption is the most appropriate to measure changes in productivity, irrespective of the underlying technology, because only the CRS assumption captures all the observed changes in productivity from one period to another.

4 Considering the small number of states compared, we have used natural clustering, based on the number of transplant beds. Analyses with data for the 2018, 2019 and 2020 produced the same clusters. Two clear clusters were identified. In cluster 1, the number of transplant beds observed in 2020 varies from 4 to 32 (with an average of 18.5 beds and a standard deviation of 7.6 beds). In cluster 2, the number of transplant beds observed in 2020 varies from 60 to 253 (with an average of 107.2 beds and a standard deviation of 64.3 beds). Cluster 1 includes the Federal District plus the following states: Bahia; Espírito Santo; Goiás; Maranhão; Mato Grosso do Sul; Pará; Paraíba; Piauí; Rio Grande do Norte; Santa Catarina. Cluster 2 includes the following states: Ceará; Minas Gerais; Paraná; Pernambuco; Rio de Janeiro; Rio Grande do Sul; São Paulo.
