# Supplementary material for: Assessing the impact of COVID-19 on the performance of organ transplant services using data envelopment analysis
Source: Health Care Manag Sci. 2023 Apr 26;26(2):217–37. doi: 10.1007/s10729-023-09637-4 (PMC10130802; doi:10.1007/s10729-023-09637-4)
Supplement: Supplementary file 1 — Supplementary file1 (DOCX 23 KB) [file 10729_2023_9637_MOESM1_ESM.docx]

# Table A - Studies of organ donation-transplantation performance assessment using DEA

| **Study** | **Method** | **DMUs** | **DEA Orientation** | **Objectives** | **Inputs** | **Outputs** |
| --- | --- | --- | --- | --- | --- | --- |
| Ozcan *et al.* (1999) | DEA | 64 OPOs in the United States | Output | Evaluate the technical efficiency of OPOs relative to optimal patterns of production in the population of OPOs in the United States | Hospital Development Formalization Index  Hospital Development FTEs (Full Time Equivalents)  Other FTEs  Operating expenses excluding Hospital Development FTE salary and fringes  Referrals | Extrarenal organs recovered  Kidneys recovered |
| Marinho and Cardoso (2007) | DEA | Years from 1995 to 2003 | Output | Evaluate changes in efficiency over time and identify trends in performance | Transplant expenses | Total number of transplants |
| Costa, Balbinotto Neto and Sampaio (2014) | DEA  MPI | Brazilian states and the Federal District in 2006 and 2011 | Output | Evaluate the efficiency of the transplant service system and change in productivity in the period | Hospital services expenses  Professional services expenses | Number of kidney transplants performed |
| Siqueira and Araujo (2018) | DEA  MPI | Brazilian states from 2013 to 2015 | Output | Evaluate the efficiency of the transplant service system and change in productivity in the period | Number of medical teams  Number of OPOs  Number of ICU beds  Number of effective donors | Number of kidney transplants from deceased donors |
| Arteaga *et al*. (2020) | DEA | 485 patients in Spain undergoing cross-over kidney transplantation from living donors | Output | Evaluate the efficiency of the procedure as well as the characteristics along which potential improvements could be introduced on a per patient basis | Age at transplant  Compatibility type  Days in dialysis  Number of previous transplants  RTX (drug)  Diabetic patient  Hypertensive patient  Smoker  Induction (type of drug used during the transplant)  CNI (drug)  mTORi (drug)  Donor age | Rejection episode  Number of rejection episodes  Graft loss  Death  Tumor development after the transplant  Number of tumor episodes in the same patient |
| Marinho and Araujo (2021) | DEA  Bootstrap  technique | Brazilian states in 2018 | Output | Evaluate the efficiency of transplant services | Number of notifications of brain death (potential donors) | Number of transplanted organs |

#

# Table B - Weight restrictions included in the study

We specified weights restrictions of the form W(p, q) ≥ 0, where p is the vector of input weights and q is the vector of output weights. For example, weigh restriction 1 (WR 1) for Model 1 establishes that the weight given to the variable Nº of transplant teams has to be equal or higher than the weight given to the variable N.º of Transplant Beds.

| **MODEL 1 - Equity in the Allocation of Resources for Transplantation** | | | | | | | | | |
| --- | --- | --- | --- | --- | --- | --- | --- | --- | --- |
|  | | Transplant waiting list {I} | | | Nº of Transplant Beds {O} | | Nºof transplant teams {O} | |  |
| WR1 | | 0 | | | -1 | | 1 | |  |
| WR2 | | 0 | | | 1000 | | -1 | |  |
| **MODEL 2 - Organ Donation and Harvest** | | | | | | | | | |
|  | Nº of ICU beds {I} | | Nº of transplant teams {I} | Nº of family consents for OD {I} | | Nº of effective donors {O} | | Donors whose organs were transplanted {O} | |
| WR1 | 0 | | -1 | 1 | | 0 | | 0 | |
| WR2 | -1 | | 0 | 1 | | 0 | | 0 | |
| WR3 | -1 | | 1 | 0 | | 0 | | 0 | |
| WR4 | 0 | | 0 | 0 | | -1 | | 1 | |
| WR5 | 0 | | 1000 | -1 | | 0 | | 0 | |
| WR6 | 1000 | | 0 | -1 | | 0 | | 0 | |
| WR7 | 1000 | | -1 | 0 | | 0 | | 0 | |
| WR8 | 0 | | 0 | 0 | | 1000 | | -1 | |

| **MODEL 3 - Organ Transplantation** | | | | | | |
| --- | --- | --- | --- | --- | --- | --- |
|  | Transplant Service Expenses {I} | Nº of Transplant Beds {I} | Nº of transplant teams {I} | Nº of effective donors {I} | Nº of Transplant Performed (AIH) {O} | Nº of patient survivors {O} |
| WR1 | 1 | -1 | 0 | 0 | 0 | 0 |
| WR2 | 1 | 0 | -1 | 0 | 0 | 0 |
| WR3 | -1 | 0 | 0 | 1 | 0 | 0 |
| WR4 | 0 | -1 | 1 | 0 | 0 | 0 |
| WR5 | 0 | 0 | 0 | 0 | -1 | 1 |
| WR6 | 1000 | 0 | 0 | -1 | 0 | 0 |
| WR7 | -1 | 1000 | 0 | 0 | 0 | 0 |
| WR8 | 0 | 1000 | -1 | 0 | 0 | 0 |
| WR9 | 0 | 1000 | 0 | -1 | 0 | 0 |
| WR10 | -1 | 0 | 1000 | 0 | 0 | 0 |
| WR11 | 0 | 0 | 1000 | -1 | 0 | 0 |
| WR12 | 0 | 0 | 0 | 0 | 1000 | -1 |

|  |
| --- |
| Note: WR = weight restriction. |

# 
